# Cytoskeletal Strains in Modeled Optohydrodynamically Stressed Healthy and Diseased Biological Cells

**DOI:** 10.1155/2012/830741

**Published:** 2012-12-05

**Authors:** Sean S. Kohles, Yu Liang, Asit K. Saha

**Affiliations:** ^1^Regenerative Bioengineering Laboratory, Department of Biology, Science Research & Teaching Center (SRTC), Portland State University, P.O. Box 751, Portland, OR 97207, USA; ^2^Department of Surgery, Oregon Health & Science University, Portland, OR 97239, USA; ^3^Center for Allaying Health Disparities through Research and Education (CADRE) and Department of Mathematics & Computer Science, Central State University, Wilberforce, OH 45384, USA

## Abstract

Controlled external chemomechanical stimuli have been shown to influence cellular and tissue regeneration/degeneration, especially with regards to distinct disease sequelae or health maintenance. Recently, a unique three-dimensional stress state was mathematically derived to describe the experimental stresses applied to isolated living cells suspended in an optohydrodynamic trap (optical tweezers combined with microfluidics). These formulae were previously developed in two and three dimensions from the fundamental equations describing creeping flows past a suspended sphere. The objective of the current study is to determine the full-field cellular strain response due to the applied three-dimensional stress environment through a multiphysics computational simulation. In this investigation, the multiscale cytoskeletal structures are modeled as homogeneous, isotropic, and linearly elastic. The resulting computational biophysics can be directly compared with experimental strain measurements, other modeling interpretations of cellular mechanics including the liquid drop theory, and biokinetic models of biomolecule dynamics. The described multiphysics computational framework will facilitate more realistic cytoskeletal model interpretations, whose intracellular structures can be distinctly defined, including the cellular membrane substructures, nucleus, and organelles.

## 1. Introduction

The current research on human diseases primarily focuses on the molecular, microbiological, immunological, and pathological influences. The mechanical basis of disease is now often being explored to decipher any direct contributions toward the physiological response [1, 2]. In functionally loaded tissues such as cartilage and bone, cells (chondrocytes and osteocytes) experience multiaxial forces (hydrostatic, compressive, tensile, and shear), which play a significant role in modulating the biological function through maintenance of the phenotype and production of a neotissue [3]. Conversely, abnormal mechanical forces (either static or dynamic) can lead to altered cell behavior resulting in pathological matrix synthesis, increased catabolic activity (degradation), and ultimately osteoarthritis or osteoporosis (apoptosis) [4]. Our previous investigations have indicated that chondrocytes and likely other cell types respond to their stress-strain environments in a temporal and spatial manner [5]. It has also just been shown that individual cellular mechanical properties may indicate the regenerative potential of mesenchymal stem cells [6]. Investigations of the biomechanics at the cellular level have also identified the biomarkers of disease. Cytoskeletal stiffness of metastatic cancer cells was reported as more than 70% softer than the benign cells that line the body cavity in patients with suspected lung, breast, and pancreatic cancer [7]. These approaches highlight the utility of single-cell biomechanics as a critical component of advancing microscale therapeutics. 

 Advancements in laser technology and microfluidics now allow the use of optical tweezers or traps and fluid mechanics to manipulate isolated single cells [8, 9]. Isolated loads can be applied experimentally to single cells in culture to quantify cellular and cytoskeletal biomechanics. One can then apply forces and displacements as small as pico-Newtons and nanometers, respectively [10–12]. The local microenvironment can therefore be precisely manipulated to facilitate biomechanical test sequences on individual biological cells and molecular structures.

 In order to explore the connection between external mechanical stimulation and cellular regeneration or degeneration, we developed a three-dimensional, multiphysics computational model to fully characterize a unique micromechanical environment. The applied stress state within our custom-fabricated optical and hydrodynamic (optohydrodynamic) trap have been mathematically developed from the fundamental equations describing microfluidic creeping flows past a suspended sphere. The objective described in the following paper is to explore the full-field cellular strain response to a range of applied stresses and cellular moduli. The described computational framework will now allow us to develop more realistic cellular models, whose intracellular structures are distinctly identified. This approach is specifically focused on addressing our ongoing efforts in health disparity research [13].

## 2. Methods

### 2.1. Single Cell Biomechanics and Optohydrodynamic Trapping

Living non-adhered, suspended osteoblasts, chondrocytes, fibroblasts, and myoblasts have recently been isolated and mechanically manipulated [12, 16]. Primary cultures of chondrogenic and osteogenic tissues were generated directly from rat long bones, while muscle cells were acquired from the mouse-derived myoblast C2C12 cell line (ATCC, CRL-1772, Manassas, VA, USA). All cells were tested at room temperature experiments (~20.5°C) in a flow media consisting of a physiological buffer resulting in a media viscosity of ~1 mPa s. This single cell biomechanical manipulation was made available by combining optical trapping with microfluidics to create the optohydrodynamic trap. This work was facilitated by an instrument, which integrates two laser-based techniques for the mechanical characterization of cellular and biomolecular structures [8, 12]. 

**Figure 1:**
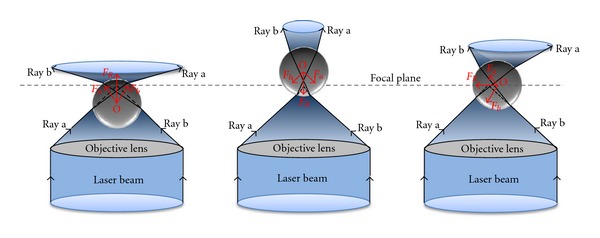
Qualitative illustration of the induced (*F*_*a*_, *F*_*b*_) and resultant (*F*_*R*_) forces created from isolated refracted light rays (a, b) driving the centroid of the cell (O) back to the focal plane associated with the high aperture objective lens.

The optical tweezers or the trap component of the device utilizes an infrared laser (*λ* = 1,064 nm) to suspend micron-sized objects with nanometer position control and pico-Newton constraining forces. In the Mie regime, where objects are larger in dimension than the wavelength of the trapping laser (here biological cells), a ray optics description indicates the transfer of refracted light and the associated momentum into trapping forces (Figure 1). Micron-particle image velocimetry can be engaged by incorporating two frequency-doubled lasers (*λ* = 532 nm) aligned through the same optical path as the OT for full-field flow velocity characterization. However, the nanoparticles associated with velocity imaging have proven deleterious to cellular health, but provide useful experimental validation of flows around synthetic micron-sized particles.

**Figure 2:**
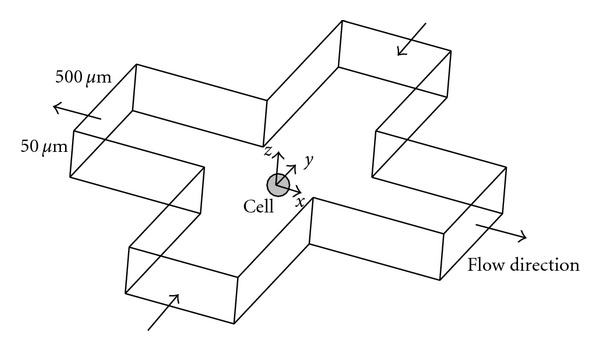
Schematic of the cross-junction microfluidic channel creating a fluid flow environment and the hydrodynamic multiaxial loading of an optically trapped biological cell [12, 14, 15].

**Figure 3:**
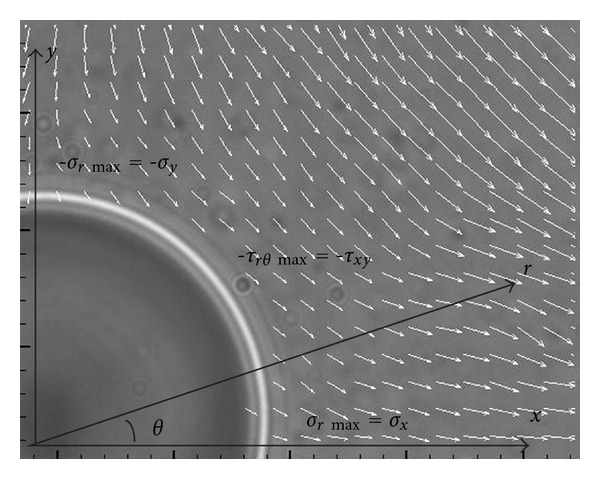
Quarter section image of an optically and hydrodynamically trapped glass bead with the surrounding fluid velocity vectors defined experimentally by the particle image velocimetry [16]. Analytically characterized flow velocity gradients are able to impart controlled normal and shear stresses onto trapped biological cells [12]. The relationship between the Cartesian and polar coordinates is indicated here within the *x*-*y* and *r*-*θ* planes, respectively.

 The hydrodynamic component of this approach is facilitated through a microfluidic chip design configured in the form of a cross-junction channel (Figure 2). This geometry creates an extensional flow environment and a stagnation point at the channel's geometric center. Cells are positioned at the centroid with the optical trap and manipulated with microfluidics, thus creating the optohydrodynamic trap. The cell experiences a total drag force equal to zero, confirmed by integrating the stress tensor as defined by the normal (form drag) and shear (friction drag) stresses (Figure 3). This reflects the mechanical stability or the saddle-point nature of the optohydrodynamic trap. Previous studies describe chip fabrication in detail including the control of the gravity-driven flow initially associated with microfluidic manipulation [8].

### 2.2. Applied Fluid Stress Analysis

 The two- and three-dimensional stress states were previously developed as applied to the surface of a nonrotating spheroid cell of radius *a*, within the optohydrodynamic trap [2, 12, 14, 15]. Briefly, the full-field fluid velocity vector **u** was constructed from the constitutive equations describing a non-rotating sphere suspended in a general linear flow with viscosity *μ* and pressure distribution *p* [17]. In the polar-spherical components (*r*-*θ*-*ϕ* magnitudes and **e**_*r*_-**e**_*θ*_-**e**_*ϕ*_ vectors), the generalized flow field produces the individual velocity components:

(1) u=U(ra−52(ar)2+32(ar)4)sin2ϕcos⁡2θer+U(ra−(ar)4)sinϕcos⁡ϕcos⁡2θeϕ+U(ra−(ar)4)sinϕcos⁡2θeθ

including the pressure distribution

(2) $$\begin{array}{c} p=p_{\infty }-\frac{5\mu U}{a}{\left(\frac{a}{r}\right)}^{3}\text{si}{\text{n}}^{2}\phi \cos2\theta . \end{array}$$

The velocity gradients can be converted into the applied fluid stresses by applying the constitutive equation for an incompressible, Newtonian fluid [18]:

(3) $$\begin{array}{c} T=-pI+2\mu E, \end{array}$$

where **T** is the stress tensor and **I** is the identity matrix associated with the local isotropic (hydrostatic) pressure distribution *p*. The strain rate tensor **E** can be characterized by the flow velocity gradient tensor and its transpose:

(4) $$\begin{array}{c} E=\left(\frac{1}{2}\right)\left[\nabla u+\nabla u^{T}\right]. \end{array}$$

By incorporating the velocity and pressure fields into the gradient analysis and then in turn into the constitutive equation, the fluidic stress tensor can be fully defined as

(5) $$\begin{array}{c} T_{rr}=-p_{\infty }+\frac{\mu U}{a}\left(2+15{\left(\frac{a}{r}\right)}^{3}-12{\left(\frac{a}{r}\right)}^{5}\right)\text{si}{\text{n}}^{2}\phi \cos2\theta e_{r},\\ T_{r\phi }=\frac{\mu U}{a}\left(2-5{\left(\frac{a}{r}\right)}^{3}+8{\left(\frac{a}{r}\right)}^{5}\right)\sin\phi \cos\phi \cos2\theta e_{\phi },\\ T_{r\theta }=-\frac{\mu U}{a}\left(2-5{\left(\frac{a}{r}\right)}^{3}+8{\left(\frac{a}{r}\right)}^{5}\right)\sin\phi \sin2\theta e_{\theta }. \end{array}$$

Defining the stress tensor at the cellular surface, *r* = *a*, produces the volumetric fluidic stress state applied to the cell:

(6) $$\begin{array}{c} t_{r}=Te_{r}=\sigma_{rr}e_{r}+\tau_{r\phi }e_{\phi }+\tau_{r\theta }e_{\theta }, \end{array}$$

where the full-field stress state can then be defined in terms of distinct normal, *σ*_*rr*_, and shear, *τ*_*rθ*_ and *τ*_*rθ*_, stress components, respectively, in polar spherical coordinates:

(7) $$\begin{array}{c} \sigma_{rr}=-p_{\infty }+\frac{5\mu U}{a}\text{si}{\text{n}}^{2}\phi \cos2\theta ,\\ \tau_{r\phi }=\frac{5\mu U}{a}\sin\phi \cos\phi \cos2\theta ,\\ \tau_{r\theta }=-\frac{5\mu U}{a}\sin\phi \sin2\theta . \end{array}$$

**Figure 4:**
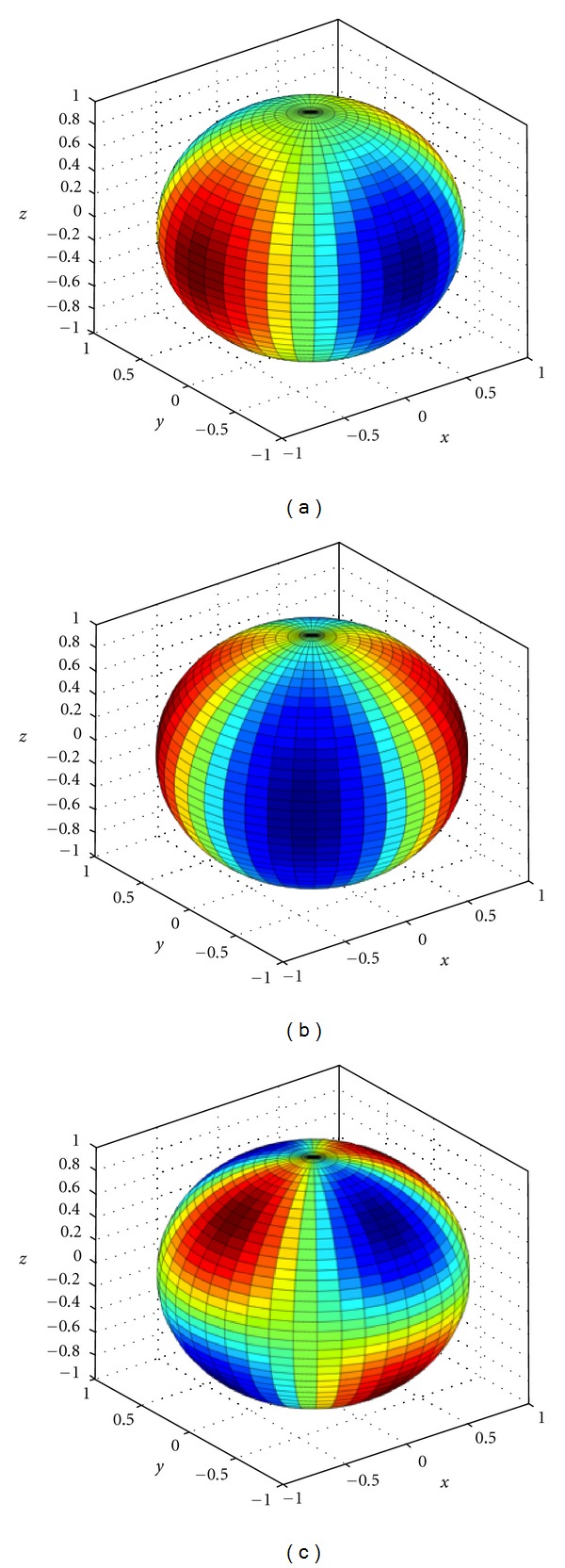
Three-dimensional representation of the applied (a) normal stresses, (b) *x*-*y* and *r*-*θ* shear stresses, and (c) *y*-*z* and *r*-*ϕ* shear stresses [14, 15] based on the original derived equations (7). Maximum positive stress levels are indicated in dark red while maximum negative stresses are in dark blue.

A three-dimensional presentation of the stresses was developed (MATLAB, MathWorks, Inc., Natick, MA) for demonstration of the site-specific nature of the stress distributions (Figure 4).

### 2.3. Stress versus Strain Modeling

 The six deviatoric stresses were combined as an effective stress value, *σ*_eff_ [19], as a means to model the three-dimensional stress state:

(8) σeff=12[(σx−σy)2+(σy−σz)2+(σz−σx)2  +6(τxy2+τyz2+τzx2)]1/2.

Here, the maximum polar coordinate derived stresses were converted to the Cartesian coordinate stresses such that the maximum normal stresses are located along the *x*-*y*-*z* axes and the maximum shear stresses are defined along the 45° orientation off-axis locations.

 Volumetric strain, *e*, based on the strain invariants, *I*_*i*_, was defined to encompass the full-field deformation response of the cell within the multiaxial fluidic loading environment and does not apply the small-displacement theory assumption. The Cartesian axes associated with the maximum normal strains were determined from a Mohr's circle analysis [12, 20]:

(9) $$\begin{array}{c} e=I_{1}+I_{2}+I_{3}=\varepsilon_{x}+\varepsilon_{y}+\varepsilon_{z}+\varepsilon_{x}\varepsilon_{y}+\varepsilon_{y}\varepsilon_{z}+\varepsilon_{z}\varepsilon_{x}+\varepsilon_{x}\varepsilon_{y}\varepsilon_{z}. \end{array}$$

The experimental approach described earlier is a planar-wise measurement technique; thus the optical depth strain value, *ε*_*z*_, can be determined through a transposition of the planar loading scenario, again through a Mohr's circle analysis [21]:

(10) $$\begin{array}{c} \varepsilon_{z}=-\frac{\nu }{1-\nu }\left(\varepsilon_{x}+\varepsilon_{y}\right). \end{array}$$

However, in this modeling presentation here, the full-field stress state and the corresponding strains are fully characterized.

 The optohydrodynamic deviatoric stress state was applied to an isotropic, homogenous biological cell (20 *μ*m in diameter) within a multiphysics computational environment (COMSOL v4.0, Palo Alto, CA, USA) in order to determine the individual principal strains and in turn the volumetric strains.

## 3. Results

**Figure 5:**
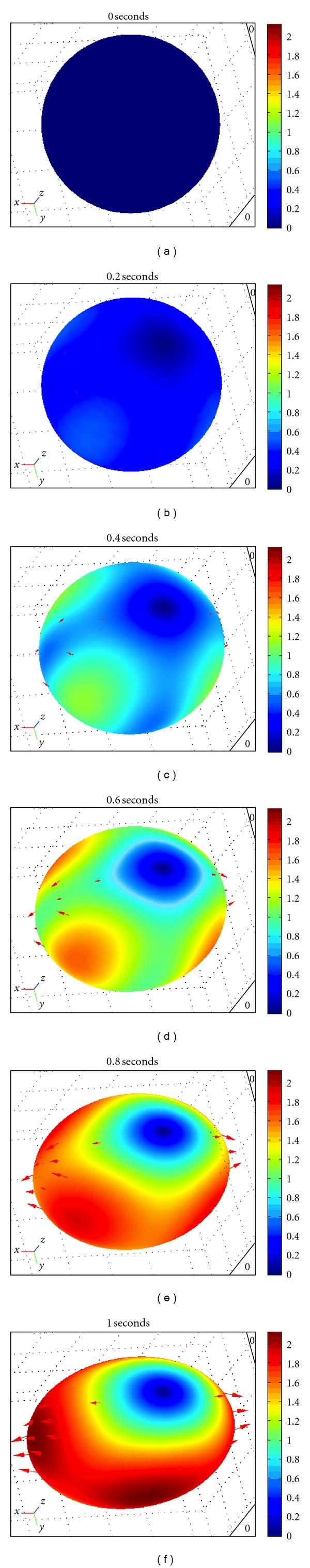
Step wise, time-sequenced volumetric cytoskeletal deformations due to applied microfluidic stresses created within an optohydrodynamic trap environment. Simulated deformations were modeled with a multiphysics computational software (COMSOL v4.0). Minimum deformation is indicated by dark blue while maximum deformation is in dark red. Units for the deformation scale are in micrometers (*μ*m).

**Figure 6:**
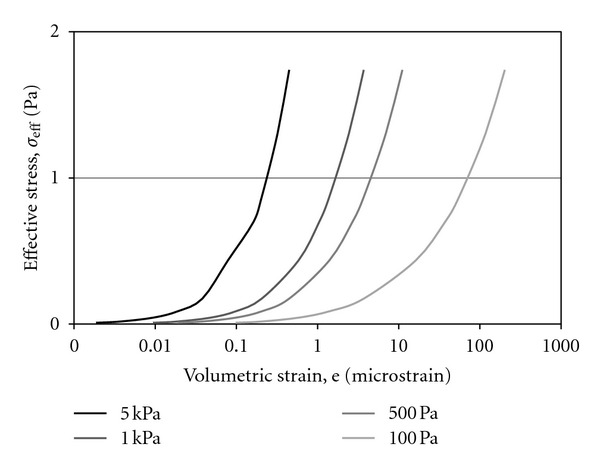
Graphical plot of the effective stresses versus volumetric strains (logarithmic scale) in modeled biological cells with varying linear elastic moduli representing the range in diseased to normal cells (low to high moduli, resp.). The modeled environment characterizes the fluid-induced stresses derived from flow velocities of *U* = 10 to 2,000 *μ*m/s within the optohydrodynamic trap.

**Figure 7:**
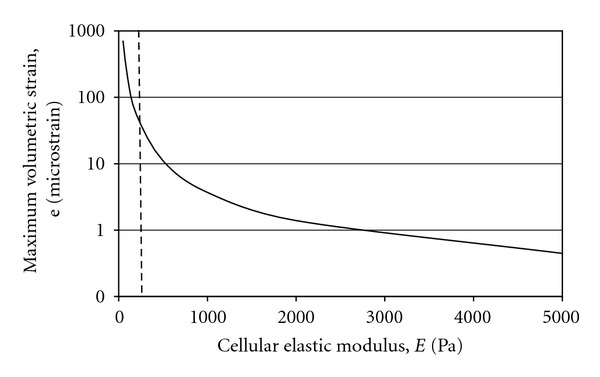
Graphical plot of the relationship between the cellular elastic moduli and the maximum volumetric strains created with a modeled flow velocity of *U* = 2,000 *μ*m/s. The dashed line indicates the hypothetical distinction between the load response for soft and stiff cells, a biomechanical response that may exacerbate the degrading health and weakened mechanical state of diseased cells during functional loading.

 In this work, we continue to examine the cellular biomechanics induced within a controlled micromechanical environment. Three-dimensional cellular strains were computationally modeled *in silico* with multiphysics software applying the analytically defined stress state (Figure 5). Volumetric stress and strain relationships indicated both the nonlinear response of the spherical cellular structure as well as the logarithmic increase in strain with subsequently softer cellular moduli (Figure 6). This biomechanical softening represents our hypothesized transition from healthy to diseased cellular states. This relationship is further explored when examining the direct relationship of strain with elastic moduli (Figure 7). The extreme strain response induced in diseased cells indicates their further vulnerability when passively or actively resisting applied stresses.

## 4. Discussion

 We explored the full-field cellular strain response to a range of applied hydrodynamic stresses and cellular moduli, representing various degrees of functional loading and health/disease, respectively. The computational framework now allows us to develop more realistic cellular models with intracellular and membranous structures, distinct in spatial, elastic, and active transport characteristics [22].

 The mechanical properties of a single cell are often formulated using either macroscopic or microscopic approaches [23]. Macroscopic approaches, as partially described in this work, homogenize every cellular component to produce an isotropic or anisotropic yet homogeneous continuum model so that the mechanical properties of cells can be formulated using temporal and spatially continuous partial differential equations [24]. Future efforts will incorporate a more microscopic approach, which regards the cell as a biocomposite material consisting of randomly or uniformly spaced anisotropic reinforcement cytoskeletal materials within an isotropic medium. The microscopic approach generally obtains the biomechanical response of a single cell by applying the mechanical boundary conditions at the individual fiber and matrix level, scaling “up” to the cellular level. Microscopic approaches often provide much more detail into the subtle interaction between the cytoskeletal fibers and matrix, which potentially leads to a more accurate model of the cellular behavior, such as characterizing the irreversible deformation of the cellular skeleton [25]. Unfortunately, refined microscopic models suffer from inhibitory computational and storage costs [26, 27].

 When interpreting the potential geometric changes in cellular shape associated with mechanical loading, the cell may experience some interesting membrane transitions triggering unique biologic cues. Under suspension and hydrostatic loading, the cell's volume (*V*) can be defined as a sphere, *V* = 3/4*πabc*, with radii *a* = *b* = *c*. As seen here during the hydrodynamic extensional loading state, the isotropic, homogenous cell model deformed into a scalene ellipsoid (*a* ≠ *b* ≠ *c*) with equal and opposite tensile and compressive strains in the horizontal plane. However, with the future inclusion of intracellular structures and organelles as well as nonlinear elastic properties assigned to the membrane and cytoskeleton, cellular models may also deform into either an oblate spheroid (formed when an ellipse is rotated about its minor axis, *a* = *b* > *c*) or a prolate spheroid (formed when an ellipse is rotated about its major axis, *a* = *b* < *c*). The resulting shape here replicated a scalene ellipsoid likely due to different maximum stresses applied in the three orthogonal axes. Ongoing experiments and modeling will continue to explore multiaxial single-cell biomechanics as well as the biologic triggers associated with geometric shape-shifting [28].

 The described multiphysics computational framework will facilitate more realistic cytoskeletal model interpretations, whose intracellular structures can be distinctly defined, including the cellular membrane substructures, nucleus, and organelles. Future results will provide mathematical outcomes supporting the ongoing investigations in tissue and cellular engineering.
